# Assessing the Biodegradation of Vulcanised Rubber Particles by Fungi Using Genetic, Molecular and Surface Analysis

**DOI:** 10.3389/fbioe.2021.761510

**Published:** 2021-10-18

**Authors:** R. Andler, V. D’Afonseca, J. Pino, C. Valdés, M. Salazar-Viedma

**Affiliations:** ^1^ Escuela de Ingeniería en Biotecnología, Centro de Biotecnología de los Recursos Naturales (Cenbio), Universidad Católica del Maule, Talca, Chile; ^2^ Centro de Investigación de Estudios Avanzados del Maule, Vicerrectoría de Investigación y Postgrado, Universidad Católica del Maule, Talca, Chile; ^3^ Laboratorio de Genética y Microevolución, Facultad de Ciencias Básicas, Universidad Católica del Maule, Talca, Chile

**Keywords:** laccase, peroxidase, *Pleurotus ostreatus*, rubber biodegradation, rubber recycling, *Trametes versicolor*, vulcanized rubber

## Abstract

Millions of tonnes of tyre waste are discarded annually and are considered one of the most difficult solid wastes to recycle. A sustainable alternative for the treatment of vulcanised rubber is the use of microorganisms that can biotransform polymers and aromatic compounds and then assimilate and mineralise some of the degradation products. However, vulcanised rubber materials present great resistance to biodegradation due to the presence of highly hydrophobic cross-linked structures that are provided by the additives they contain and the vulcanisation process itself. In this work, the biodegradation capabilities of 10 fungal strains cultivated in PDA and EM solid medium were studied over a period of 4 weeks. The growth of the strains, the mass loss of the vulcanised rubber particles and the surface structure were analysed after the incubation period. With the white rot fungi *Trametes versicolor* and *Pleurotus ostreatus*, biodegradation percentages of 7.5 and 6.1%, respectively, were achieved. The FTIR and SEM-EDS analyses confirmed a modification of the abundance of functional groups and elements arranged on the rubber surface, such as C, O, S, Si, and Zn, due to the biological treatment employed. The availability of genomic sequences of *P. ostreatus* and *T. versicolor* in public repositories allowed the analysis of the genetic content, genomic characteristics and specific components of both fungal species, determining some similarities between both species and their relationship with rubber biodegradation. Both fungi presented a higher number of sequences for laccases and manganese peroxidases, two extracellular enzymes responsible for many of the oxidative reactions reported in the literature. This was confirmed by measuring the laccase and peroxidase activity in cultures of *T. versicolor* and *P. ostreatus* with rubber particles, reaching between 2.8 and 3.3-times higher enzyme activity than in the absence of rubber. The integrative analysis of the results, supported by genetic and bioinformatics tools, allowed a deeper analysis of the biodegradation processes of vulcanised rubber. It is expected that this type of analysis can be used to find more efficient biotechnological solutions in the future.

## Introduction

The automotive industry has generated an enormous amount of tyre waste, where landfill is not a viable or sustainable alternative (Bowles et al., 2020). In nature, we can find biological degradation mechanisms carried out by microorganisms with the capacity to use a wide range of compounds such as natural polymers. However, tyre waste has a highly complex structure, preventing effective natural biodegradation processes. Since only 20–25% w/w of a tyre corresponds to natural rubber and the remaining percentage to a mixture of synthetic rubber, carbon black, antioxidants, accelerators, retardants, elemental sulphur, among other compounds (Stevenson et al., 2008), cell colonisation and, consequently, biodegradation is inhibited. In addition to the highly resistant chemicals as part of the tyre, the high degree of crosslinking and the low or no reactivity of the functional groups in the tyre structure make the mixing of this residue with other materials a major challenge (Andler, 2020; Simon-Stőger and Varga, 2021).

Several tyre-recycling processes have been described, and most of them are focused on the modification of the chemical structure and, thus, achieving devulcanisation. These include chemical processes using different peroxides (Rooj et al., 2011; Sabzekar et al., 2015; Hejna et al., 2019), physical processes such as grinding, ultrasound, microwaves or thermo-mechanical processes (Feng and Isayev, 2006; Wang et al., 2013; Formela and Cysewska, 2014; Garcia et al., 2015; Asaro et al., 2018; Formela et al., 2019; Li et al., 2020) and biological processes using microorganisms, mainly bacteria (Tatangelo et al., 2016; Ghavipanjeh et al., 2018; Aboelkheir et al., 2019; Kaewpetch et al., 2019).

The biodegradation of unvulcanised rubber, such as natural rubber, latex or poly (*cis*-1, 4-isoprene), has previously been studied in detail. Those studies pointed out that the main microorganisms responsible for performing polymer cleavage into oligomers are actinomycetes belonging to the genera *Gordonia*, *Streptomyces*, *Nocardia*, *Actinoplanes*, among others (Jendrossek et al., 1997). The metabolic pathways involved during the oxidation of oligo (*cis*-1,4-isoprene) molecules by *β*-oxidation (Hiessl et al., 2012) and cell cultures, using the polymer as sole carbon source, have also been described (Andler et al., 2018b). Furthermore, the enzymes responsible for the first oxidative attack, the so-called “rubber oxygenases,” have been studied biochemically (Jendrossek and Birke, 2019) and have been used for *in vitro* applications (Andler et al., 2018a, 2020). However, biodegradation studies using vulcanised rubber are scarce, and much remains to be understood.

Fungi are characterised by their biochemical and ecological capacity to degrade contaminants of different nature and varied chemical structures (Harms et al., 2011). They present a series of unique characteristics for the biodegradation of pollutants or toxic substances, including their ability to extend through substrates because of their filamentous structure, powerful enzyme production system and the capacity to produce natural surfactants that favour the degradative process (Sánchez, 2020). The biodegradation of synthetic polymers using fungi has been extensively studied using polyethylene terephthalate (PET), polyethylene (PE), polypropylene (PP), polyvinyl chloride (PVC) and polystyrene (PS) plastics. While commercial plastics also contain additives that impart the physicochemical properties of interest to the final product and prolong the life of plastic products (Hahladakis et al., 2018), the amount of these additives relative to the polymer is considerably lower when compared to the amount of additives present in tyres (Altenhoff et al., 2019).

Given the similarity between lignocellulosic biomass and the additives present in tyres, with many of them being aromatic compounds, white-rot fungi are excellent candidates for detoxifying tyre waste. In a previous study, the degradative capacity of 15 fungi was analysed using the additive Poly R-478 as a model aromatic compound. The authors found that the fungi *Pleurotus sajor-caju*, *Trametes versicolor* and *Resinicium bicolor* had a higher incidence of decolorising the additive and maintained their extracellular enzymatic activity after cultivation (Bredberg et al., 2002).

Despite the important degradative capacity of fungi, given their high metabolic rate and tremendous capacity for the degradation and mineralisation of various environmental pollutants, there is little information regarding their potential use as catalysts for rubber wastes. In this study, we analysed the effects generated on the surface of vulcanised rubber particles after incubation with different fungal species and their linkage with the possible enzymes responsible for these modifications, using molecular and bioinformatics tools.

## Materials and Methods

### Rubber Particles

Rubber particles (RP) were purchased from Trelleborg AB, Germany. For all experiments performed, a particle size between 1 and 2 mm was used. To avoid contamination, rubber particles were washed with 70% ethanol, dried at 80°C for 24 h and sterilised at 121°C for 20 min in separate aluminium envelopes.

### Growth Experiments

Ten different fungal species, namely *Coriolus multicolor*, *Gonnoderma aplanatum*, *Lentinula edodes*, *Lenzites trabea*, *Lenzites betulinus*, *Pleurotus ostreatus*, *Pleurotus eryngii*, *Postia placenta*, *Stereum hirsutum* and *Trametes versicolor*, were studied. The strains were incubated at 28°C using plates with potato dextrose agar (PDA) and malt extract agar (MEA). After obtaining a fully grown plate, 0.5 g of rubber particles was added to each plate under sterile conditions and incubated for 4 weeks. Each experiment was performed in triplicate.

### Recovery of Rubber Particles

After the incubation period, the rubber particles were carefully collected from the agar plate under sterile conditions and washed with 70% ethanol and 10% sodium hypochlorite until total removal of the fungi. Subsequently, the solvent was removed, and the rubber particles were dried at 50°C for 72 h. Control samples were treated under the same conditions to prevent the potential oxidative effect of sodium hypochlorite from affecting subsequent analyses.

### Mass Reduction Measurement

The mass of the rubber particles was analysed before and after the incubation period with the fungi. The loss of mass was calculated as follows:

$$\%\mathrm{weight}\mathrm{reduction}=\frac{\left(m_{i}-m_{f}\right)}{m_{f}}\cdot 100,$$ (1)

where m_i_ is the initial mass of the rubber particles and m_f_ is the final mass of the rubber particles.

### FTIR Analysis

10 mg of dried rubber particles were analysed by attenuated total reflectance (ATR-FTIR). The infrared spectra were obtained using an FTIR spectrometer (Agilent, Cary 630) with the ATR technique. Absorbance was measured in the wavelength range of 400–40,00 cm^−1^, with a resolution of 4 cm^−1^. For each sample, 64 scans were performed, and the background was subtracted using the Agilent MicroLab PC software.

### Scanning Electron Microscopy

An electron microscope TESCAN VEGA 3 with probe EDS BRUKER QUANTAX was used. The method was based on the ASTM E1508 standard “Standard Guide for Quantitative Analysis by Energy-Dispersive Spectroscopy”. Samples were covered by a cathodic spray system with Palladium Gold in the Hummer 6.2 equipment.

### Enzyme Activity Assay

Liquid cultures of *T. versicolor* and *P. ostreatus* were performed by cutting three 1 × 1 cm^2^ plugs from fully grown PDA plates and poured into 250-ml shake flasks with 50 ml of potato dextrose broth (PDB). Sterile rubber particles were incorporated to the flasks while the corresponding controls did not contain rubber particles. Cultivations were performed at 30 °C, 100 rpm for 5 days. Supernatants were obtained by centrifugation at 10,000 × g for 20 min at 4°C and used for enzyme activity measurements.

Laccase and peroxidase activities were quantified spectrophotometrically (Agilent, Cary 100 Bio) at 415 nm by detecting the oxidation of 2,20-azino-bis(3-ethylbenzothiazoline-6-sulfonate) (ABTS). For laccase activity, 100 μL of sample were mixed with a solution containing 900 μL of 10 mM ABTS and 0.2 M sodium acetate pH 5.0. For peroxidase activity, 100 μL of sample were mixed with a solution containing 800 μL of 10 mM ABTS and 100 M sodium acetate pH 5.0 and 100 μL of 20 mM H_2_O_2_.

### Genomic Data Collection of Fungal Species

Two Basidiomycetes species were selected in this study as sample sets of the biodegradation of vulcanised rubber. Publicly available genomic assemblies were downloaded from the Joint Genome Institute’s fungal genome portal MycoCosm (Grigoriev et al., 2014), JGI Genome Portal - unified access to all JGI genomic datasets (Mukherjee et al., 2021) and NCBI Genome Assembly (Schoch et al., 2020).

### Bioinformatic Analysis

Two specific enzymes (laccase EC 1.10.3.2 and manganese peroxidase EC 1.11.1.13) were searched in sequences deposited in a public database for the 10 fungal species analysed in this study. The database used consisted of identical protein groups of the National Center for Biotechnology Information (www.ncbi.nlm.nih.gov).

For analysis of the similarity at the amino acid level among the sequences of laccases and manganese peroxidase, we used the MEGA-X (Kumar et al., 2018) roand T-COFFEE (https://coffee.crg.cat/apps/tcoffee/) (Notredame et al., 2000) software packages with the default parameters. To generate the protein alignment figures, the ESPript 3.0 software (https://espript.ibcp.fr/) (Robert and Gouet, 2014) was used, highlighting the regions with higher similarity among the sequences: black represents 100% similarity, grey represents 90 to 80% and white similarity below 70%. Each analysis used sequences of laccases and manganese peroxidase separately from *Pleurotus ostreatus* and *Trametes versicolor.*

To evaluate the presence of a conserved domain among the sequences of laccases and manganese peroxidase from *P. ostreatus* and *T. versicolor*, we used the Conserved Domain Database (CDD) from the NCBI (https://www.ncbi.nlm.nih.gov/Structure/cdd/cdd.shtml) and PFAM (http://pfam.xfam.org/) programmes (Lu et al., 2020)*.*

## Results and Discussion

### Fungal Growth in the Presence of Vulcanised Rubber Particles

**FIGURE 1:**
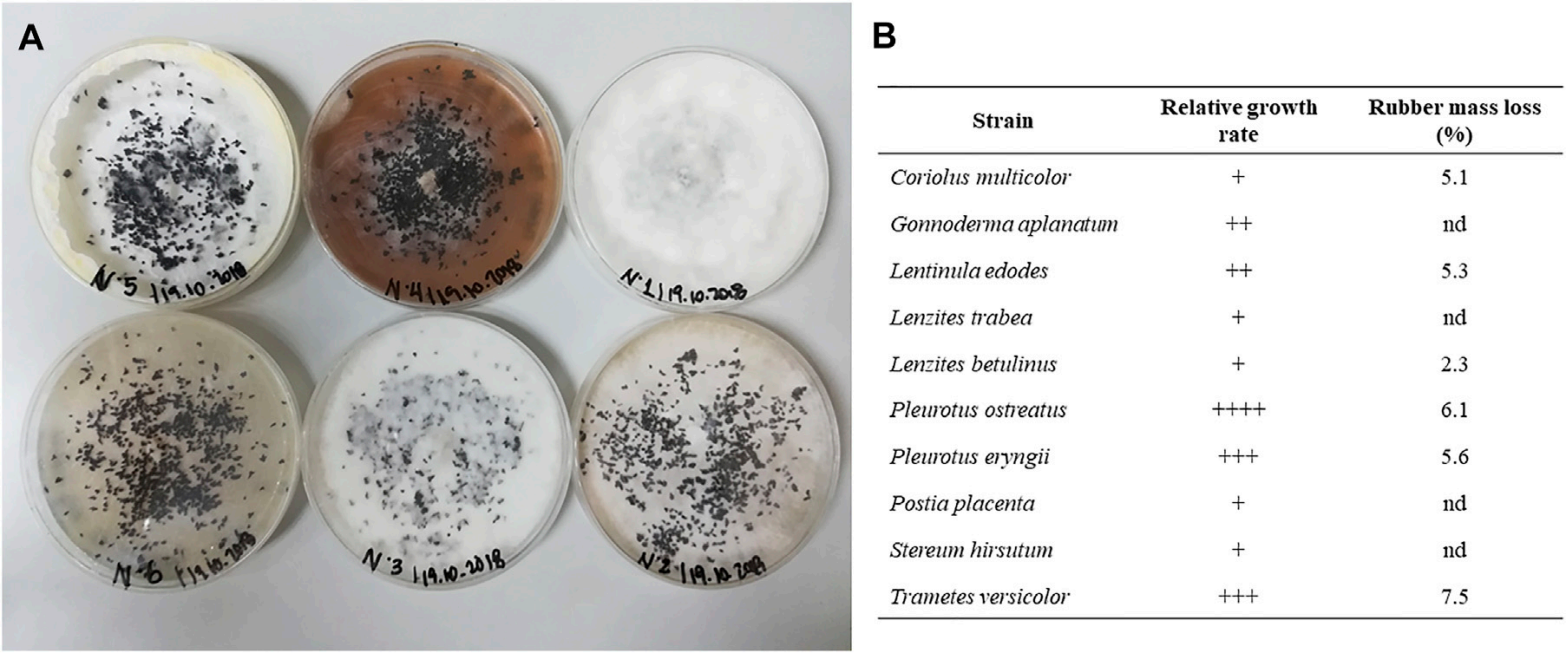
**(A)** Growth of fungal species in the presence of vulcanised rubber particles. **(B)** Relative growth rate and rubber mass reduction of the 10 fungal species after incubation.

The fungal strains were evaluated in terms of radial growth when incubated in PDA and EM media in Petri dishes, and no significant differences were found between the culture media used (data not shown). Figure 1A shows some of the fungi incubated with the vulcanised rubber particles already incorporated. There was a different fungus-rubber interaction depending on the fungal strain used. Three different behaviours stand out, namely total coating, partial coating and zero coating. Of the 10 strains analysed, only *P. ostreatus* and *T. versicolor* achieved a total coating of the vulcanised rubber particles, whereas the strains *C. multicolor*, *L. edodes*, *L. betulinus* and *P. eryngii* showed partial coating. The time required to reach a fully-grown plate was different for each fungus, which was qualitatively analysed by the relative growth rate (Figure 1B). Based on this, *P. ostreatus* obtained complete growth in the shortest time (between 24 and 30 h), followed by *P. eryngii* and *T. versicolor* (from 36 to 42 h). In general, the observations of the level of coating were related to the percentage mass loss of the vulcanised rubber particles at the end of the incubation periods, while the relative growth rate was not always a precise indicator. The highest mass loss was 7.5 ± 0.3%, obtained after cultivation with *T. versicolor*, followed by 6.1 ± 0.4% obtained with *P. ostreatus*. Based on these results, vulcanised rubber particles treated with *T. versicolor* and *P. ostreatus* strains were selected for surface analysis.

Bredberg et al. (2002) studied the detoxification of vulcanised rubber by different wood-rotting fungi, using the aromatic polymeric dye polyvinylamine sulfonate anthrapyridone (Poly-R478) as a model compound. The authors showed that only three strains were able to biodegrade Poly-R478, namely *Pleurotus sajor-caju*, *T. versicolor* and *Recinicium bicolor*. Subsequently, they used the treated rubber in cultures with *Acidithiobacillus ferrooxidans* and reached higher growth rates compared to rubber without fungal treatment. It was assumed that the extracellular enzymes from the selected fungi degraded aromatic structures and that the rubber obtained had a reduced toxicity. As in our study, only the cultures with white rot fungi obtained positive results.

### Surface Analysis of Rubber Particles After Fungal Incubation

**FIGURE 2:**
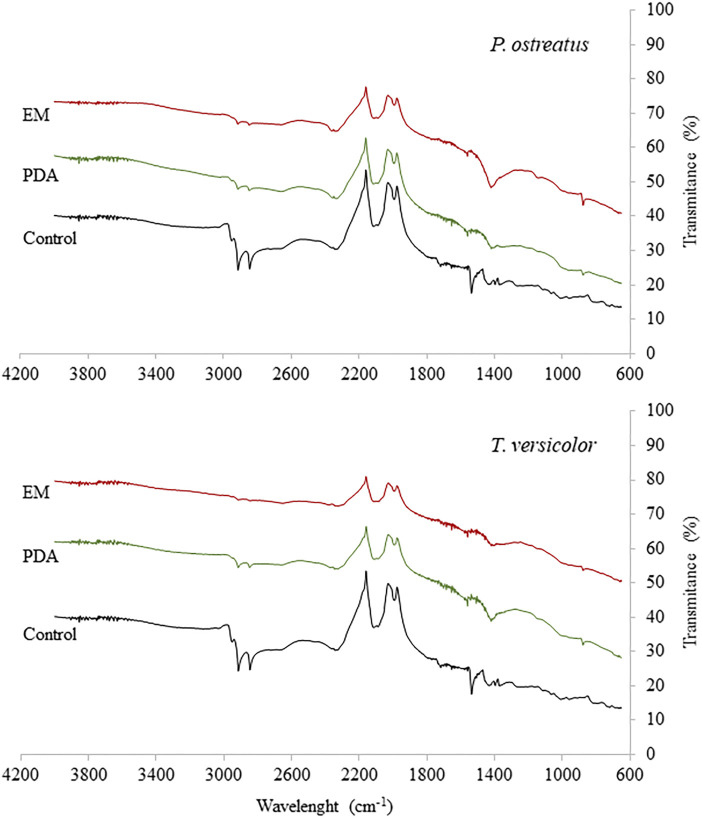
FTIR spectrum of vulcanised rubber particles after incubation with *Trametes versicolor* and *Pleurotus ostreatus* using PDA and EM medium.

Changes in the rubber surface were analysed by FTIR (Figure 2). The functional groups observed may indicate the presence of compounds such as black carbon, aromatic oils, antioxidants, accelerators, among others (Altenhoff et al., 2019). Doublet was observed between 2,926 and 2,853 cm^−1^ (stretching of CH_2_), which was drastically reduced after the treatment, with the greatest reduction in *T. versicolor* with EM medium and *P. ostreatus* with EM medium (Gorassini et al., 2016). A loss of a signal was also observed at approximately 1,715 cm^−1^ in the treatment with *T. versicolor* on EM and PDA media and for *P. ostreatus* on EM and PDA media, corresponding to carbonyl groups (C=O), which are typical of the rubber chain (Gorassini et al., 2016). A band at approximately 1,550 cm^−1^, associated with carboxylate or conjugated ketone, was totally lost after cultivation of both fungal species (Stelescu et al., 2017). After incubation with *T. versicolor*, a small band close to 1,455 cm^−1^, assigned to C-H bending of CH_2_, was maintained in PDA, but it decreased using EM. However, for *P. ostreatus,* the band decreased for the assay with PDA and increased significantly in the treatment with EM. Most likely, the sporulation of the fungus was influenced when PDA medium was used, and therefore, the enzymatic metabolism involved in the decrease of the band was influenced by the sporulation process stimulated by PDA (Su et al., 2012). We detected a variation in the polymer structure after treatments corresponding to alkene groups (–C=CH) at a wavelength of 874 cm^−1^. This band was not present in the control, but it appeared after incubation with *T. versicolor* and *P. ostreatus* on both media (Colom et al., 2016). One reason for this may be the exposure of groups related to isoprene and butadiene present in rubber, which, before treatment, were hidden in more interior areas of the material.

These results show that the treatment using *T. versicolor* with EM allowed a greater transformation of the surface of the rubber material, suggesting that the EM medium favours the degradation in a better way than PDA for *T. versicolor*. For *P. ostreatus*, the PDA medium allowed, in general, a greater decrease in bands versus the control. The EM medium could stimulate the generation of secondary metabolites, allowing a more efficient degradation of rubber compared to the type of metabolites stimulated by the PDA medium.

**FIGURE 3:**
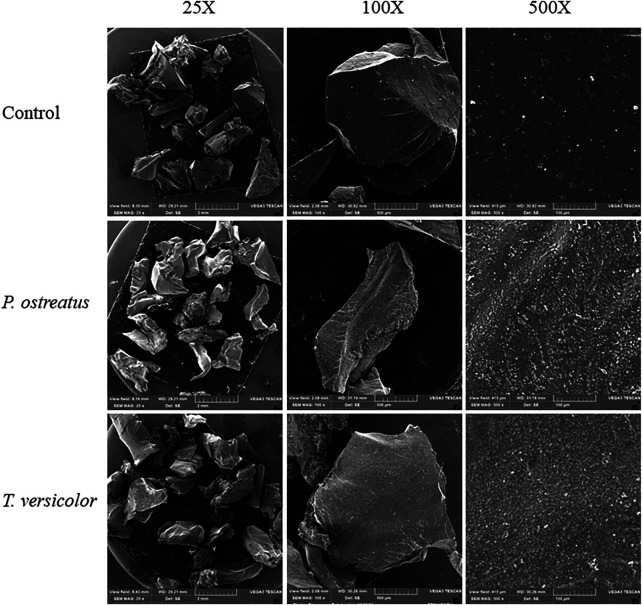
SEM-EDS analysis of vulcanised rubber particles.

We used SEM analysis to demonstrate the changes in the rubber surface against mechanical, chemical or biological treatments (Li et al., 2012; Aboelkheir et al., 2019). In this study, SEM analysis revealed morphological changes of the analysed rubber surface after biological treatments (*P. ostreatus* and *T. versicolor* using PDA medium) in comparison with the control at 500x (Figure 3). In both treatments, particle size was approximately 500 to 1,200 µm and was therefore not influenced by the treatments, in contrast to a previous study (Li et al., 2012). The size reported in this work is compatible with treatments with lower-performance grinding. When comparing the control versus the biological treatments, an increase in the roughness of the material was observed, which was similar for *T. versicolor* and *P. ostreatus*. After the treatment, the surface showed cracks, facilitating the breaking of the rubber particles by subsequent treatments, such as mechanical ones, since they can spread rapidly, generating fractures (Kroon, 2011). This result is also related to the elemental analysis of C, O, S, Si, and Zn on the rubber surface (Table 2), where the carbon content decreased in the rubber particles subjected to the treatments. The opposite was observed for O and S; it should be noted that the PDA medium lacks sulphates, and therefore, the increase in S and O is not a result of the culture medium. We therefore recommend that the rubber particles were washed with ethanol and a solution of chlorine before superficial analysis (see Methodology), and therefore, the increases in Si and Zn after the biological treatment are not due to contamination, as both elements are commonly present in tyres (Adathodi et al., 2018; Altenhoff et al., 2019). Zinc is used in the non-accelerated vulcanisation of rubber, whereas silicium is used as a filler (Rattanasom et al., 2007). With *P. ostreatus*, a higher percentage of Si was detected compared to *T. versicolor*, whereas for Zn, the percentage was higher with the use of *T. versicolor*. The changes at the surface can be attributed to oxidation-reduction reactions by the enzymes secreted by the microorganisms, exposing a different proportion of the elements initially arranged on the surface. It is suggested that the observed changes were due to enzymatic processes related to the assimilation of microorganisms towards the components present on the surface of the treated material, which is corroborated by the loss of rubber mass after treatment.

### Laccase and Peroxidase Activity

**FIGURE 4:**
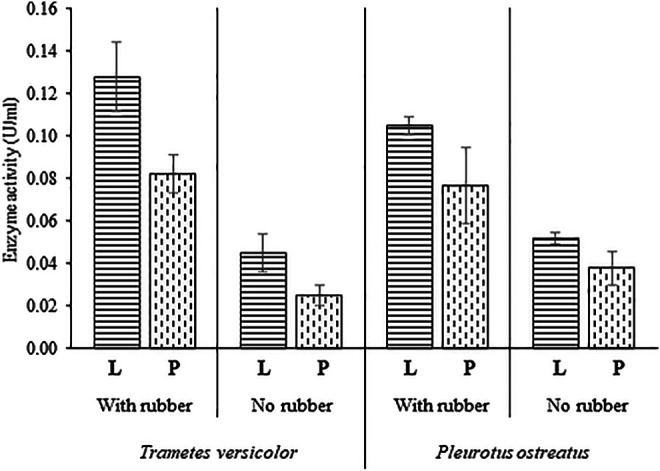
Laccase and peroxidase activity in cultures of *Trametes versicolor* and *Pleurotus ostreatus* in the presence and the absence of vulcanized rubber particles. L, laccasse, P, peroxidase. Measurements were carried out in triplicates.

Laccase and peroxidase activities were detected in all the conducted experiments (Figure 4). In cultures of *T. versicolor*, the presence of rubber particles revealed a 2.8-fold increase of laccase activity and 3.3-fold increase of peroxidase activity compared to the cultures without rubber particles. Likewise in cultures of *P. ostreatus*, laccase and peroxidase activities increased by double when rubber particles were in the cultivation medium. A maximum laccase activity of 0.128 U ml^−1^ was calculated for *T. versicolor* cultures, a 18% more than for *P. ostreatus* cultures. An enhanced laccase and peroxidase activity can be achieved by the addition of different mediators into the cultivation medium such as xylidine, ferulic acid, veratrylalcohol, pyrogallol and copper (Vranska et el. 2016), enzymes like feruloyl esterase, aryl-alcohol oxidase, quinone reductases, lipases, catechol 2, 3-dioxygenase (Kumar and Chandra 2020) or by culture media optimization (Wang et al., 2014; Hahn Schneider 2018). The increase of the laccase and peroxidase activity in the cultures of *T. versicolor* and *P. ostreatus* predicts the affinity of these ligninolytic enzymes for aromatic substrates present in the rubber composition.

The presence of laccase and peroxidase enzymes has been described during the degradation process of natural rubber by the bacteria *Bacillus subtilis* (Nayanashree and Thippeswamy, 2015). Nevertheless, the enzymatic mechanism is still unknown and rubber oxygenases (Lcp, RoxA, RoxB) have been well characterized for the cleavage of natural rubber (Andler et al., 2018a; Jendrossek and Birke, 2020). The complexity of working with multi-components substrates as rubber is to reveal which of those parts are directly related to the activity of the extracellular enzymes. The current work has shown that the presence of rubber act as an inducer of laccase and peroxidase activities, and provides a more concrete result in the role of these groups of enzymes for vulcanized rubber biodegradation. However, it would be necessary to study the effect of laccases and peroxidases with each of the different components of vulcanized rubber to gain a deeper understanding of the reaction mechanisms.

### Genomic Insights Into the Pathways for Rubber Degradation

Obtaining genome data is the initial step in understanding the biology of *P. ostreatus* and *T. versicolor*. Fungi present a wide variety of functional proteins that play different roles in the acquisition of nutrients and protection against nearby organisms or unfavourable environmental conditions (Kim et al., 2016).

The genomes of *P. ostreatus* and *T. versicolor* have been sequenced, and an analysis of the published fungal genomes, sourced from the public database (Grigoriev et al., 2014; Schoch et al., 2020; Mukherjee et al., 2021), revealed that the *P. ostreatus* haploid genome contains 11 chromosomes, with a length of 34.3 Mbp and a G + C content of ∼51%. For *T. versicolor*, there is no information in the public database about the number of chromosomes; however, the total genomic size is 44.8 Mb, with a G + C content of ∼58% (Table 1). Although there is a large variation in the genome size in fungi, the average genome size of fungal species taken during this study was 40.0 Mb. Fungal species harbour small genomes with highly specific genic regions and reduced non-coding regions (Galagan et al., 2003; Martinez et al., 2004). Fungi belonging to the phylum Basidiomycota have an average genome size of 46 Mb (Mohanta and Bae, 2015). In line with this, the sequenced genomes of *Pleurotus* taxa were assembled to less than 50 M bp but exhibited large numbers of annotated protein-coding genes and few constituent TEs (Table 1). However, as mentioned before, there were genome size variations among the two species, which could have arisen from unequal TE variations. The number of transposable elements (TEs), including retrotransposons and DNA transposons, accounted for approximately 253 of the *P. ostreatus* genome and 233 of the *T. versicolor* genome. For *P. ostreatus*, it was possible to determine notable genomic differences in the regions corresponding to TEs, where 80 identified TE families represented 2.5–6.2% of the genome sizes (Téllez-Téllez and Díaz-Godínez, 2019). Transposable elements are undoubtedly an important source of genetic variation in fungi, as previously found for other fungal species (Labbé et al., 2012).

**TABLE 1:** General features of the genome of *P. ostreatus* and *T. versicolor*.

Feature	*Pleurotus ostreatus*	*Trametes versicolor*
Chromosomes	11	N/A
Genome size (Mbp)	34,3	44,79
GC content, %	50.85	57.7
DNA scaffolds	12 and 572	283
Total No. TE	253	233
Genes total number	12,330	14,572
Protein coding genes	12,330	14,302
Protein coding genes with function prediction	706	N/A
Protein coding genes with enzymes	1,677	N/A
Non coding genes	315	234
Pseudo genes	3	2

Considering the coding gene sequences in fungi, on average, the Basidiomycota group encodes for 15,431.51 genes in their genomes. The average numbers of annotated genes are 12,330 for *P. ostreatus* per genome and 14,572 for the *T. versicolor* genome (Table 1). For the noncoding gene, we found scaffolds of the genome assembly, with 315 and 234 genes, respectively. There were three and two pseudogenes predicted, corresponding to 0.24% of the genome assembly of *P. ostreatus* and 0.014% of the genome assembly of *T. versicolor*.

Comparative analysis of fungal genomes showed that fungi are highly divergent. The *P. ostreatus* genome sequence assembly was distributed across of 12 and 572 scaffolds. Of these, 56 were smaller than 1 kb (Alfaro et al., 2016; Castanera et al., 2016). The genome of *T. versicolor* comprised only 283 scaffolds. The average protein coding genes in *P. ostreatus* were 12,330, with 14,302 for *T. versicolor*. These coding genes have homologues with known proteins deposited in the NCBI nr, Pfam, SwissProt and TrEMBL databases.

Both fungal species can partially degrade vulcanised rubber, and genome data revealed that both of fungi encode for a large set of enzymes involved in the degradation of rubber materials. The protein coding genes with enzymes for *P. ostreatus* included 1,677 genes (Table 1)*.* For *T. versicolor*, such data were not available in the public repositories. However, based on a previous study, *T. versicolor* has an expansion of the AA2 gene family (26 genes), a feature that is also found in the central polyporoid clade (Miyauchi et al., 2018). The putative peroxidase genes (PoPOD) were obtained from the Joint Genome Institute (JGI) (Mukherjee et al., 2021). The gene density and the mean size of the protein-coding genes in both species of fungi used in this study secrete different enzymes, among which are laccases, manganese peroxidases, versatile peroxidases, glycosylhydrolases, peptidases and fungal esterases/lipases (Ruiz-Dueñas et al., 2011). These enzymes can degrade complex compounds such as lignin as well as certain industrial pollutants contaminating vulcanised rubber. Despite the advances in comparative genomics, the genera *Pleurotus* and *Trametes* are under-exploited, and a variety of potential biotechnological applications in different industries can be elucidated based on their genomes.

### Bioinformatic Analysis for Rubber Bioremediation

#### Laccase and Manganese Peroxidase in the Fungi Genomes

The genomic data of several species evaluated in this work revealed information concerning the presence of laccases and manganese peroxidase enzymes. For the species *L. edodes*, *L. betulinus*, *P. eryngii*, *P. ostreatus*, *P. placenta*, *S. hirsutum* and *T. versicolor*, database searches revealed the presence of several sequences of laccase already sequenced; the species *L. edodes*, *P. ostreatus*, *T. versicolor* and *P. eryngii* showed the highest number of laccase sequences (Table 2). In addition, the species *G. applanatum*, *L. edodes*, *P. eryngii*, *P. ostreatus*, *S. hirsutum* and *T. versicolor* also presented several sequences of manganese peroxidase already characterised. Species such as *L. edodes*, *P. ostreatus* and *T. versicolor* presented the highest number of characterised enzymes of manganese peroxidase in the evaluated database (Table 2). In our experiments, these species showed a high ability to degrade rubber. The high number of deposited sequences of laccase and manganese peroxidase for these species could reinforce their role as microorganisms that can degrade rubber and other polymers naturally, using laccase and manganese peroxidase enzymes (Nayanashree and Thippeswamy, 2015). However, the species *C. multicolor* and *L. trabea* presented no sequences of both mentioned enzymes. Also, *G. applanatum* had no deposited sequence of laccase; *L. betulinus* and *P. placenta* had no sequences of manganese peroxidase in the database used in this study*.*

**TABLE 2:** Number of laccases and manganese peroxidase in the genomes of fungi species studied.

Fungi species	Laccase	Manganese peroxidase
*Coriolus multicolor*	NA	NA
*Ganoderma applanatum*	NA	3
*Lentinula edodes*	48	9
*Lenzites trabea*	NA	NA
*Lenzites betulinus*	7	NA
*Pleurotus eryngii*	30	1
*Pleurotus ostreatus*	44	17
*Postia placenta*	4	NA
*Stereum hirsutum*	13	2
*Trametes versicolor*	37	14

#### Conservation Among Laccases and Manganese Peroxidase Enzymes From *P. ostreatus* and *T. versicolor*

Laccase presented three multicopper oxidase-conserved domains in its structure, namely Cu-oxidase, Cu-oxidase 2 and Cu-oxidase 3. For all sequences, these domains were located in the same region. For Cu-oxidase 3 domain, its location in the sequences started around the 31 amino acid position; it had a size of around 119 amino acids. The Cu-oxidase domain started from the 160 to 169 amino acid position and presented about 159 amino acids. The Cu-oxidase 2 domain started in the 366 to 388 amino acid position and generally had a size of 137 amino acids. These domains can use copper ions as cofactors to oxidise a broad range of substrates (Nayanashree and Thippeswamy, 2015; Arregui et al., 2019).

Manganese peroxidase has two conserved domains: a peroxidase domain with 229 amino acids and another domain, the so-called “peroxidase extension region,” with a size of 79 amino acids. The peroxidase domain in the sequences starts from 49 to 53 amino acid position and the peroxidase extension region from 279 to 286 amino acid position. These enzymatic domains are involved in oxidative reactions that use hydrogen peroxide as the electron acceptor (Nayanashree and Thippeswamy, 2015; Wang et al., 2016; Arregui et al., 2019).

For alignment at the amino acid level of laccase sequences from *P. ostreatus*, 18 sequences were used, and from *T. versicolor*, 23 sequences were used. All partial sequences were removed from the alignment analysis. Laccases from *P. ostreatus* showed a size of around 530 amino acids and a different pattern of the amino acid content; they were divided into four group of laccases. For *T. versicolor*, the laccases showed an average size of 520 amino acids and showed the same behaviour as those from *P. ostreatus* regarding the amino acid content; they were divided into three groups. In the evaluated database, the information about the specific name of each laccase in both species was inconsistent, making it difficult to predict the correct name per group. However, based on the amino acid alignment and the similarity, the laccase groups were determined.

In *P. ostreatus*, the groups of alignment represented for A and B presented high similarity among their amino acid contents of the studied sequences; however, for the groups C and D, the sequences were almost identical (see Supplementary Figure S1 online). In *T. versicolor*, group A also presented a high similarity among the amino acid contents of the sequences, and groups B and C were almost identical (see Supplementary Figure S2 online).

Regarding manganese peroxidase, we also evaluated its amino acid conservation among all sequences deposited in the NCBI database. Both species presented manganese peroxidase with a size of around 360 amino acids. For *P. ostreatus*, we evaluated 11 sequences and for *T. versicolor* 10 sequences. Similar to the laccase analysis, all partial sequences were removed from the alignment analysis. The species *P. ostreatus* presented four groups based on the amino acid contents of the sequences (see Supplementary Figure S3 online). The groups A, B and C were almost identical, and group D of manganese peroxidase presented high similarity regarding the amino acid content. The species *T. versicolor* had two groups of manganese peroxidases enzymes (see Supplementary Figure S4 online), presenting high similarity among the sequences for the group representing A, while for the group B, the sequences are almost identical*.* When generating an alignment using all sequences together, one analysis for laccase and another analysis for manganese peroxidase, the level of amino acid similarity decreased (data not shown). Only separated regions of conserved domains were maintained. These results indicate the presence of different isoforms of laccases and manganese peroxidase in the genomes of these two studied fungi or the lack of genomic information in the accessed database (Yuan et al., 2016).

## Conclusion

Sustainable solutions for the management of toxic solid wastes, such as vulcanised rubber, are of great importance, and the use of microorganisms has great potential. However, the hydrophobic nature and the large amounts of additives present in the material prevent high biodegradation rates. In this study, we analysed the degradation potential of different fungal strains, finding two white rot fungi that stood out, namely *T. versicolor* and *P. ostreatus*. The effects of biological treatments in terms of surface modifications and the presence of laccase and peroxidase activity were linked to genomic and bioinformatic analyses. We found a strong relationship between the percentage of biodegradation of rubber particles and the availability of laccase and manganese peroxidase enzymes in these species, two of the main enzymes used in the bioremediation of xenobiotics. The results obtained are promising, highlighting white rot fungi as potent biodegrading agents. Among the most interesting considerations is the non-specificity of the enzymatic attack provided by these fungi, which is necessary in highly recalcitrant materials such as tyre waste. Given the complexity of the biodegradation of vulcanised rubber, integrated and comprehensive studies are required to achieve a deeper understanding and to postulate new biotechnological processes for more effective biodegradation or biotransformation.

## Data Availability

The original contributions presented in the study are included in the article/Supplementary Material, further inquiries can be directed to the corresponding author.
