# Supplementary material for: Assessing the Biodegradation of Vulcanised Rubber Particles by Fungi Using Genetic, Molecular and Surface Analysis
Source: Front Bioeng Biotechnol. 2021 Oct 18;9:761510. doi: 10.3389/fbioe.2021.761510 (PMC8558253; doi:10.3389/fbioe.2021.761510)
Supplement: Supplementary file 1 [file DataSheet2.PDF]

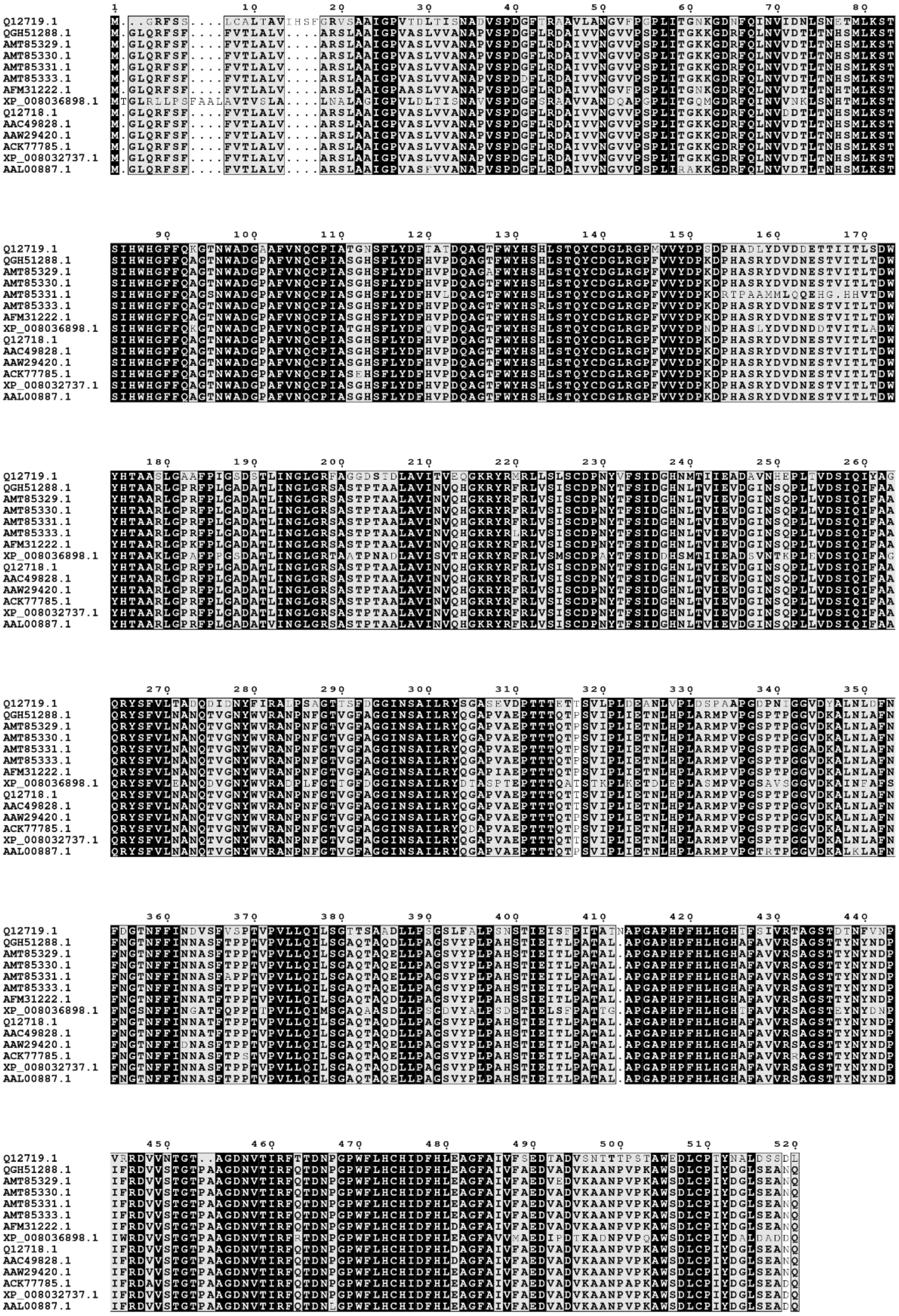
Q12719.1
QGH51288.1
AMT85329.1
AMT85330.1
AMT85331.1
AMT85333.1
AFM31222.1
XP_008036898.1
Q12718.1
AAC49828.1
AAW29420.1
ACK77785.1
XP_008032737.1
AAL00887.1

a

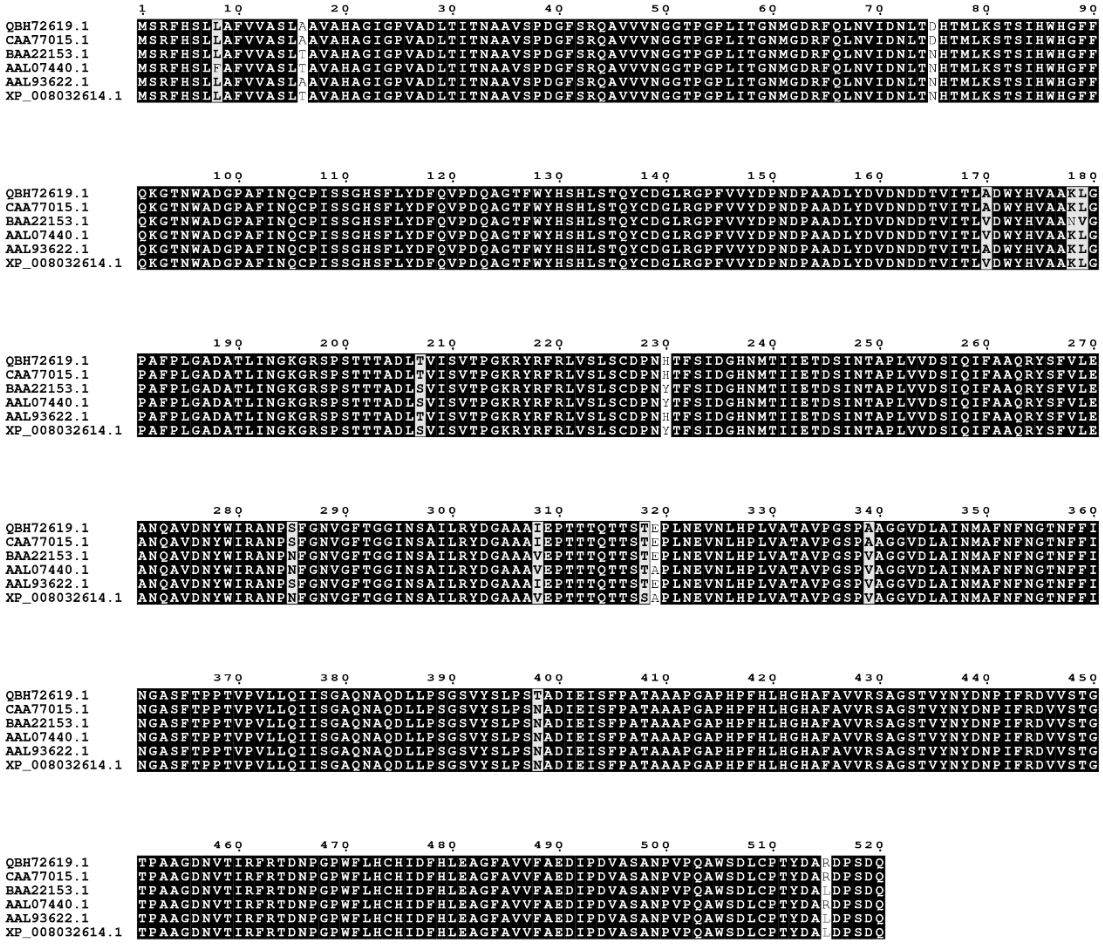
1 10 20 30 40 50 60 70 80 90
QBH72619.1 MSRFHSLLAFVVASLAAVAHAGIGPVADLTITNAAVSPDGFSRQAVVVNGGTPGPLITGNMGDRFQLNVIDNLTDHTMLKSTSIHWHGFF
CAA77015.1 MSRFHSLLAFVVASLAAVAHAGIGPVADLTITNAAVSPDGFSRQAVVVNGGTPGPLITGNMGDRFQLNVIDNLTDHTMLKSTSIHWHGFF
BAA22153.1 MSRFHSLLAFVVASLTAVAHAGIGPVADLTITNAAVSPDGFSRQAVVVNGGTPGPLITGNMGDRFQLNVIDNLTNHTMLKSTSIHWHGFF
AAL07440.1 MSRFHSLFAFVVASLTAVAHAGIGPVADLTITNAAVSPDGFSRQAVVVNGGTPGPLITGNMGDRFQLNVIDNLTNHTMLKSTSIHWHGFF
AAL93622.1 MSRFHSLLAFVVASLAAVAHAGIGPVADLTITNAAVSPDGFSRQAVVVNGGTPGPLITGNMGDRFQLNVIDNLTNHTMLKSTSIHWHGFF
XP_008032614.1 MSRFHSLLAFVVASLTAVAHAGIGPVADLTITNAAVSPDGFSRQAVVVNGGTPGPLITGNMGDRFQLNVIDNLTNHTMLKSTSIHWHGFF
100 110 120 130 140 150 160 170 180
QBH72619.1 QKGTNWADGPAFINQCPISSGHSFLYDFQVPDQAGTFWYHSHLSTQYCDGLRGPFVVYDPNDPAADLYDVDNDDTVITLADWYHVAAKLG
CAA77015.1 QKGTNWADGPAFINQCPISSGHSFLYDFQVPDQAGTFWYHSHLSTQYCDGLRGPFVVYDPNDPAADLYDVDNDDTVITLADWYHVAAKLG
BAA22153.1 QKGTNWADGPAFINQCPISSGHSFLYDFQVPDQAGTFWYHSHLSTQYCDGLRGPFVVYDPNDPAADLYDVDNDDTVITLVDWYHVAANVG
AAL07440.1 QKGTNWADGPAFINQCPISSGHSFLYDFQVPDQAGTFWYHSHLSTQYCDGLRGPFVVYDPNDPAADLYDVDNDDTVITLVDWYHVAAKLG
AAL93622.1 QKGTNWADGPAFINQCPISSGHSFLYDFQVPDQAGTFWYHSHLSTQYCDGLRGPFVVYDPNDPAADLYDVDNDDTVITLADWYHVAAKLG
XP_008032614.1 QKGTNWADGPAFINQCPISSGHSFLYDFQVPDQAGTFWYHSHLSTQYCDGLRGPFVVYDPNDPAADLYDVDNDDTVITLVDWYHVAAKLG
190 200 210 220 230 240 250 260 270
QBH72619.1 PAFPLGADATLINGKGRSPSTTTADLTVISVTPGKRYRFRLVSLSCDPNHTFSIDGHNMTIIETDSINTAPLVVDSIQIFAAQRYSFVLE
CAA77015.1 PAFPLGADATLINGKGRSPSTTTADLTVISVTPGKRYRFRLVSLSCDPNHTFSIDGHNMTIIETDSINTAPLVVDSIQIFAAQRYSFVLE
BAA22153.1 PAFPLGADATLINGKGRSPSTTTADLSVISVTPGKRYRFRLVSLSCDPNYTFSIDGHNMTIIETDSINTAPLVVDSIQIFAAQRYSFVLE
AAL07440.1 PAFPLGADATLINGKGRSPSTTTADLSVISVTPGKRYRFRLVSLSCDPNYTFSIDGHNMTIIETDSINTAPLVVDSIQIFAAQRYSFVLE
AAL93622.1 PAFPLGADATLINGKGRSPSTTTADLTVISVTPGKRYRFRLVSLSCDPNHTFSIDGHNMTIIETDSINTAPLVVDSIQIFAAQRYSFVLE
XP_008032614.1 PAFPLGADATLINGKGRSPSTTTADLSVISVTPGKRYRFRLVSLSCDPNYTFSIDGHNMTIIETDSINTAPLVVDSIQIFAAQRYSFVLE
280 290 300 310 320 330 340 350 360
QBH72619.1 ANQAVDNYWIRANPSFGNVGFTGGINSAILRYDGAAAIEPTTTQTTSTEPLNEVNLHPLVATAVPGSPAAGGVDLAINMAFNFNGTNFFI
CAA77015.1 ANQAVDNYWIRANPSFGNVGFTGGINSAILRYDGAAAIEPTTTQTTSTEPLNEVNLHPLVATAVPGSPAAGGVDLAINMAFNFNGTNFFI
BAA22153.1 ANQAVDNYWIRANPNFGNVGFTGGINSAILRYDGAAAVEPTTTQTTSTEPLNEVNLHPLVATAVPGSPVAGGVDLAINMAFNFNGTNFFI
AAL07440.1 ANQAVDNYWIRANPNFGNVGFTGGINSAILRYDGAAAVEPTTTQTTSTAPLNEVNLHPLVATAVPGSPVAGGVDLAINMAFNFNGTNFFI
AAL93622.1 ANQAVDNYWIRANPSFGNVGFTGGINSAILRYDGAAAIEPTTTQTTSTEPLNEVNLHPLVATAVPGSPVAGGVDLAINMAFNFNGTNFFI
XP_008032614.1 ANQAVDNYWIRANPNFGNVGFTGGINSAILRYDGAAAVEPTTTQTTSSAPLNEVNLHPLVATAVPGSPVAGGVDLAINMAFNFNGTNFFI
370 380 390 400 410 420 430 440 450
QBH72619.1 NGASFTPPTVPVLLQIISGAQNAQDLLPSGSVYSLPSTADIEISFPATAAAPGAPHPFHLHGHAFAVVRSAGSTVYNYDNPIFRDVVSTG
CAA77015.1 NGASFTPPTVPVLLQIISGAQNAQDLLPSGSVYSLPSNADIEISFPATAAAPGAPHPFHLHGHAFAVVRSAGSTVYNYDNPIFRDVVSTG
BAA22153.1 NGASFTPPTVPVLLQIISGAQNAQDLLPSGSVYSLPSNADIEISFPATAAAPGAPHPFHLHGHAFAVVRSAGSTVYNYDNPIFRDVVSTG
AAL07440.1 NGASFTPPTVPVLLQIISGAQNAQDLLPSGSVYSLPSNADIEISFPATAAAPGAPHPFHLHGHAFAVVRSAGSTVYNYDNPIFRDVVSTG
AAL93622.1 NGASFTPPTVPVLLQIISGAQNAQDLLPSGSVYSLPSNADIEISFPATAAAPGAPHPFHLHGHAFAVVRSAGSTVYNYDNPIFRDVVSTG
XP_008032614.1 NGASFTPPTVPVLLQIISGAQNAQDLLPSGSVYSLPSNADIEISFPATAAAPGAPHPFHLHGHAFAVVRSAGSTVYNYDNPIFRDVVSTG
460 470 480 490 500 510 520
QBH72619.1 TPAAGDNVTIRFRTDNPGPWFLHCHIDFHLEAGFAVVFAEDIPDVASANPVPQAWSDLCPTYDARDPSDQ
CAA77015.1 TPAAGDNVTIRFRTDNPGPWFLHCHIDFHLEAGFAVVFAEDIPDVASANPVPQAWSDLCPTYDARDPSDQ
BAA22153.1 TPAAGDNVTIRFRTDNPGPWFLHCHIDFHLEAGFAVVFAEDIPDVASANPVPQAWSDLCPTYDALDPSDQ
AAL07440.1 TPAAGDNVTIRFRTDNPGPWFLHCHIDFHLEAGFAVVFAEDIPDVASANPVPQAWSDLCPTYDARDPSDQ
AAL93622.1 TPAAGDNVTIRFRTDNPGPWFLHCHIDFHLEAGFAVVFAEDIPDVASANPVPQAWSDLCPTYDALDPSDQ
XP_008032614.1 TPAAGDNVTIRFRTDNPGPWFLHCHIDFHLEAGFAVVFAEDIPDVASANPVPQAWSDLCPTYDALDPSDQ

b

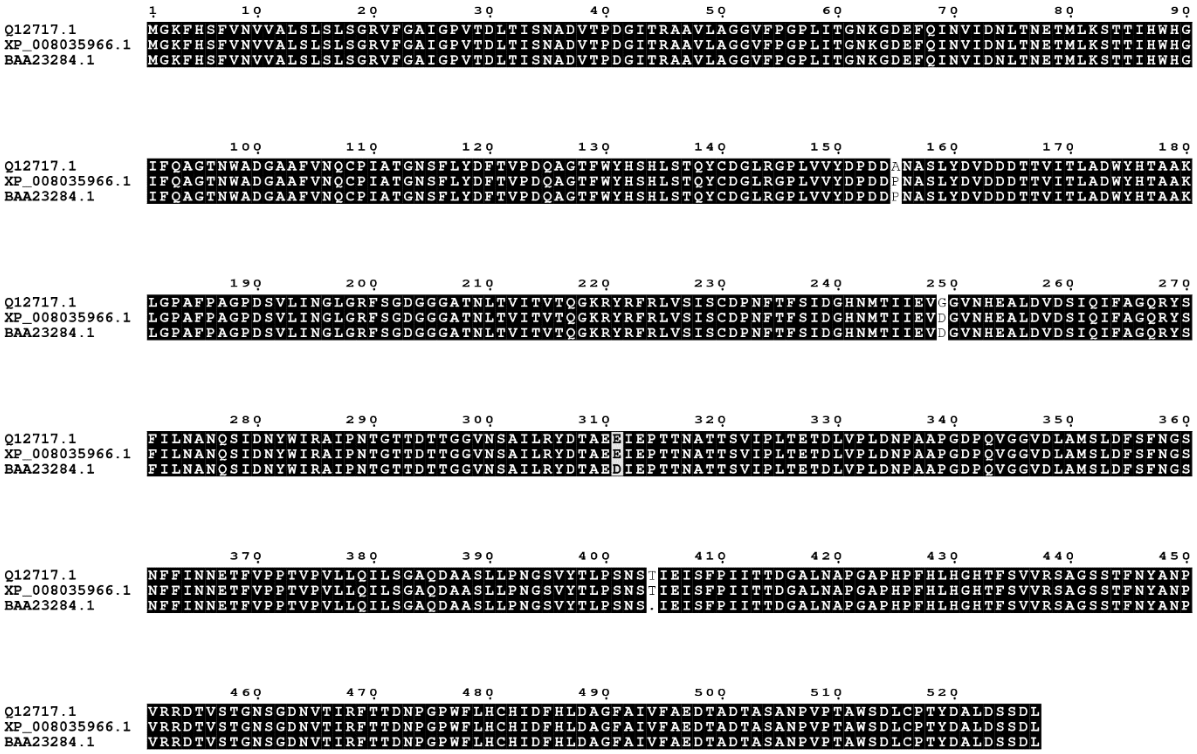

c

Supplementary Figure S2. Amino acid alignment of laccases from *Trametes versicolor*. a, b and c: different groups of laccases. Highlighted amino acid in black: 100% of similarity among the amino acids; grey: 90–80%; white: similarity under 70%.
