# Supplementary material for: Assessing the Biodegradation of Vulcanised Rubber Particles by Fungi Using Genetic, Molecular and Surface Analysis
Source: Front Bioeng Biotechnol. 2021 Oct 18;9:761510. doi: 10.3389/fbioe.2021.761510 (PMC8558253; doi:10.3389/fbioe.2021.761510)
Supplement: Supplementary file 2 [file DataSheet4.PDF]

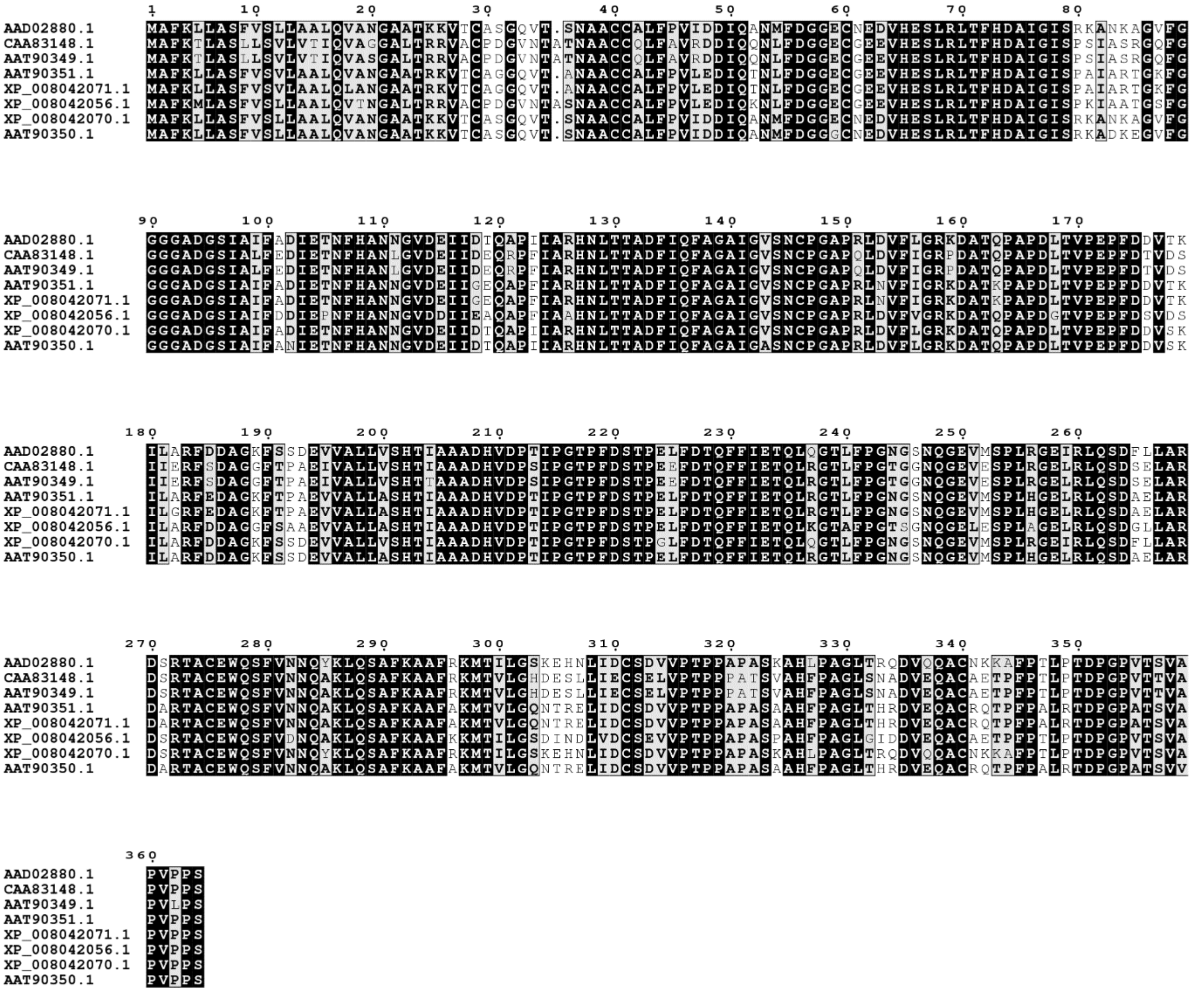
1 10 20 30 40 50 60 70 80
AAD02880.1 MAFKLLASFVSLLAALQVANGAATKKVTCASGQVT.SNAACCALFPVIDDIQANMFDGGECNEDVHESLRLTFHDAIGISRKANKAGVFG
CAA83148.1 MAFKTLASLLSVLVTIQVAGGALTRRVACPDGVNTATNAACCQLFAVRDDIQQNLFDGGECGEEVHESLRLTFHDAIGISPSIASRGQFG
AAT90349.1 MAFKTLASLLSVLVTIQVASGALTRRVACPDGVNTATNAACCQLFAVRDDIQQNLFDGGECGEEVHESLRLTFHDAIGISPSIASRGQFG
AAT90351.1 MAFKLLASFVSVLAALQVANGAATRKVTCAGGQVT.ANAACCALFPVLEDIQTNLFDGGECGEEVHESLRLTFHDAIGISPAIARTGKFG
XP_008042071.1 MAFKLLASFVSVLAALQLANGAATRKVTCAGGQVT.ANAACCALFPVLEDIQTNLFDGGECGEEVHESLRLTFHDAIGISPAIARTGKFG
XP_008042056.1 MAFKMLASFVSLLAALQVTNGALTRRVACPDGVNTASNAACCALFPVLEDIQKNLFDGGECGEEVHESLRLTFHDAIGISPKIAATGSFG
XP_008042070.1 MAFKLLASFVSLLAALQVANGAATKKVTCASGQVT.SNAACCALFPVIDDIQANMFDGGECNEDVHESLRLTFHDAIGISRKANKAGVFG
AAT90350.1 MAFKLLASFVSLLAALQVANGAATKKVTCASGQVT.SNAACCALFPVIDDIQANMFDGGGCNEDVHESLRLTFHDAIGISRKADKEGVFG
90 100 110 120 130 140 150 160 170
AAD02880.1 GGGADGSIAIFADIETNFHANNGVDEIIDTQAPIIARHNLTTADFIQFAGAIGVSNCPGAPRLDVFLGRKDATQPAPDLTVPEPFDDVTK
CAA83148.1 GGGADGSIALFEDIETNFHANLGVDEIIDEQRPFIARHNLTTADFIQFAGAIGVSNCPGAPQLDVFIGRPDATQPAPDLTVPEPFDTVDS
AAT90349.1 GGGADGSIALFEDIETNFHANLGVDEIIDEQRPFIARHNLTTADFIQFAGAIGVSNCPGAPQLDVFIGRPDATQPAPDLTVPEPFDTVDS
AAT90351.1 GGGADGSIAIFADIETNFHANNGVDEIIGEQAPFIARHNLTTADFIQFAGAIGVSNCPGAPRLNVFIGRKDATKPAPDLTVPEPFDDVTK
XP_008042071.1 GGGADGSIAIFADIETNFHANNGVDEIIGEQAPFIARHNLTTADFIQFAGAIGVSNCPGAPRLNVFIGRKDATKPAPDLTVPEPFDDVTK
XP_008042056.1 GGGADGSIAIFDDIEPNFHANNGVDDIIEAQAPFIAAHNLTTADFIQFAGAIGVSNCPGAPRLDVFVGRKDATQPAPDGTVPEPFDSVDS
XP_008042070.1 GGGADGSIAIFADIETNFHANNGVDEIIDTQAPIIARHNLTTADFIQFAGAIGVSNCPGAPRLDVFLGRKDATQPAPDLTVPEPFDDVSK
AAT90350.1 GGGADGSIAIFANIETNFHANNGVDEIIDTQAPIIARHNLTTADFIQFAGAIGASNCPGAPRLDVFLGRKDATQPAPDLTVPEPFDDVSK
180 190 200 210 220 230 240 250 260
AAD02880.1 ILARFDDAGKFSSDEVVALLVSHTIAAADHVDPTIPGTPFDSTPELFDTQFFIETQLQGTLFPGNGSNQGEVMSPLRGEIRLQSDFLLAR
CAA83148.1 IIERFSDAGGFTPAEIVALLVSHTIAAADHVDPSIPGTPFDSTPEEFDTQFFIETQLRGTLFPGTGGNQGEVESPLRGELRLQSDSELAR
AAT90349.1 IIERFSDAGGFTPAEIVALLVSHTTAAADHVDPSIPGTPFDSTPEEFDTQFFIETQLRGTLFPGTGGNQGEVESPLRGELRLQSDSELAR
AAT90351.1 ILARFEDAGKFTPAEVVALLASHTIAAADHVDPTIPGTPFDSTPELFDTQFFIETQLRGTLFPGNGSNQGEVMSPLHGELRLQSDAELAR
XP_008042071.1 ILGRFEDAGKFTPAEVVALLASHTIAAADHVDPTIPGTPFDSTPELFDTQFFIETQLRGTLFPGNGSNQGEVMSPLHGELRLQSDAELAR
XP_008042056.1 ILARFDDAGGFSAAEVVALLASHTIAAADHVDPTIPGTPFDSTPELFDTQFFIETQLKGTAFPGTSGNQGELESPLAGELRLQSDGLLAR
XP_008042070.1 ILARFDDAGKFSSDEVVALLVSHTIAAADHVDPTIPGTPFDSTPGLFDTQFFIETQLQGTLFPGNGSNQGEVMSPLRGEIRLQSDFLLAR
AAT90350.1 ILARFDDAGKFSSDEVVALLASHTIAAADHVDPTIPGTPFDSTPELFDTQFFIETQLRGTLFPGNGSNQGEVMSPLHGELRLQSDAELAR
270 280 290 300 310 320 330 340 350
AAD02880.1 DSRTACEWQSFVNNQYKLQSAFKAAFRKMTILGSKEHNLIDCSDVVPTPPAPASKAHLPAGLTRQDVQQACNKKAFPTLPTDPGPVTSVA
CAA83148.1 DSRTACEWQSFVNNQAKLQSAFKAAFRKMTVLGHDESLLIECSELVPTPPPATSVAHFPAGLSNADVEQACAETPFPTLPTDPGPVTTVA
AAT90349.1 DSRTACEWQSFVNNQAKLQSAFKAAFRKMTVLGHDESLLIECSELVPTPPPATSVAHFPAGLSNADVEQACAETPFPTLPTDPGPVTTVA
AAT90351.1 DARTACEWQSFVNNQAKLQSAFKAAFAKMTVLGQNTRELIDCSDVVPTPPAPASAAHFPAGLTHRDVEQACRQTPFPALRTDPGPATSVA
XP_008042071.1 DARTACEWQSFVNNQAKLQSAFKAAFAKMTVLGQNTRELIDCSDVVPTPPAPASAAHFPAGLTHRDVEQACRQTPFPALRTDPGPATSVA
XP_008042056.1 DSRTACEWQSFVDNQAKLQSAFKAAFKKMTILGSDINDLVDCSEVVPTPPAPASPAHFPAGLGIDDVEQACAETPFPTLPTDPGPVTSVA
XP_008042070.1 DSRTACEWQSFVNNQYKLQSAFKAAFRKMTILGSKEHNLIDCSDVVPTPPAPASKAHLPAGLTRQDVQQACNKKAFPTLPTDPGPVTSVA
AAT90350.1 DARTACEWQSFVNNQAKLQSAFKAAFAKMTVLGQNTRELIDCSDVVPTPPAPASAAHFPAGLTHRDVEQACRQTPFPALRTDPGPATSVV
360
AAD02880.1 PVPPS
CAA83148.1 PVPPS
AAT90349.1 PVLPS
AAT90351.1 PVPPS
XP_008042071.1 PVPPS
XP_008042056.1 PVPPS
XP_008042070.1 PVPPS
AAT90350.1 PVPPS

a

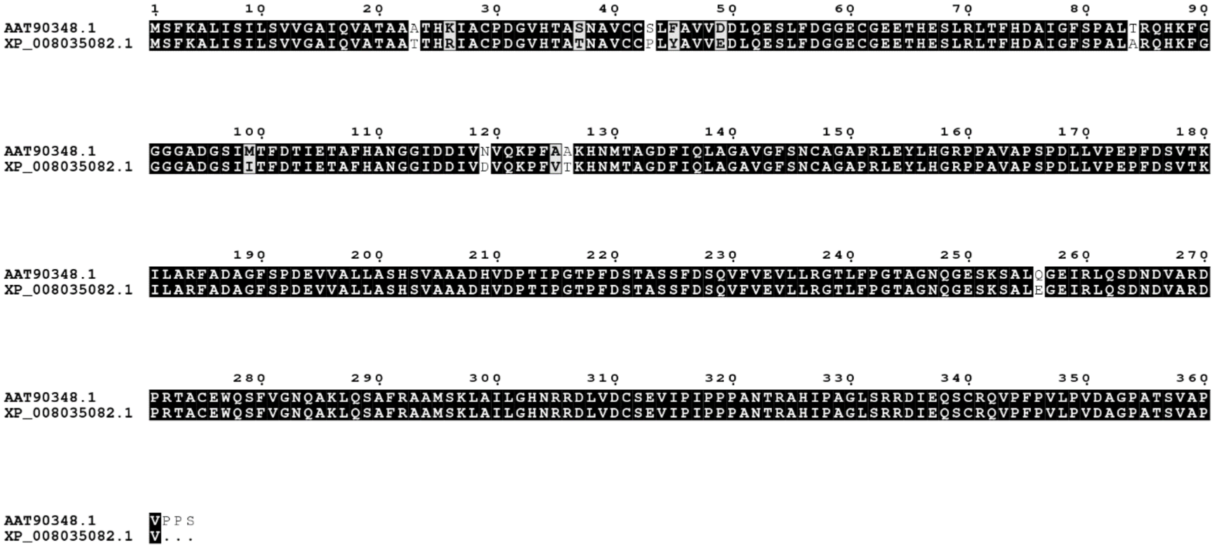

b

Supplementary Figure S4. Amino acid alignment of manganese peroxidases from *Trametes versicolor*. a and b: different groups of manganese peroxidases. Highlighted amino acid in black: 100% of similarity among the amino acids; grey: 90–80%; white: similarity under 70%.
