# Supplementary material for: Assessing the Biodegradation of Vulcanised Rubber Particles by Fungi Using Genetic, Molecular and Surface Analysis
Source: Front Bioeng Biotechnol. 2021 Oct 18;9:761510. doi: 10.3389/fbioe.2021.761510 (PMC8558253; doi:10.3389/fbioe.2021.761510)
Supplement: Supplementary file 3 [file DataSheet3.PDF]

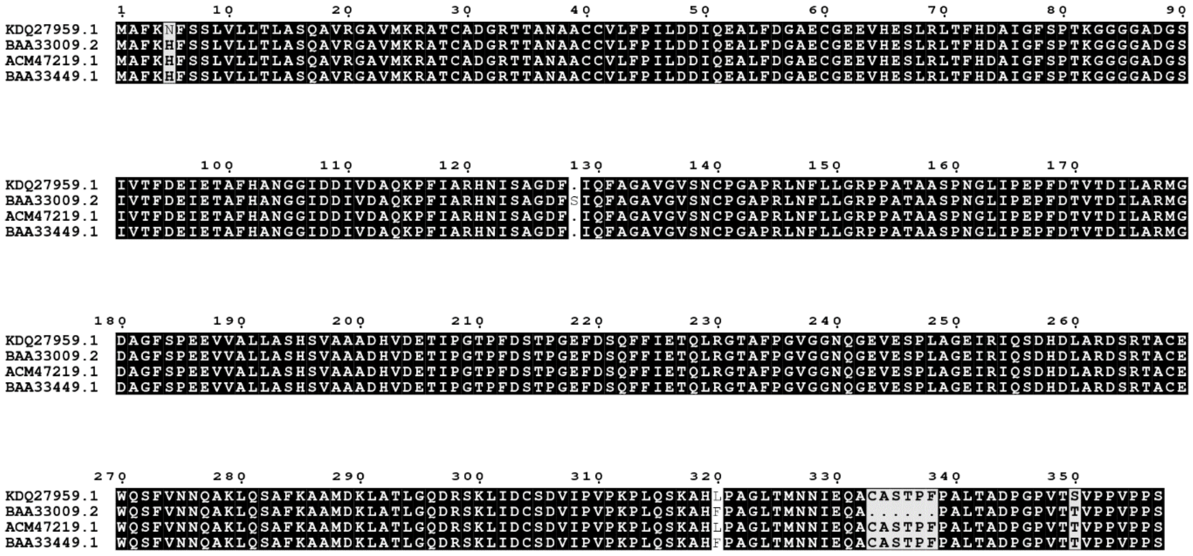
KDQ27959.1
BAA33009.2
ACM47219.1
BAA33449.1

a

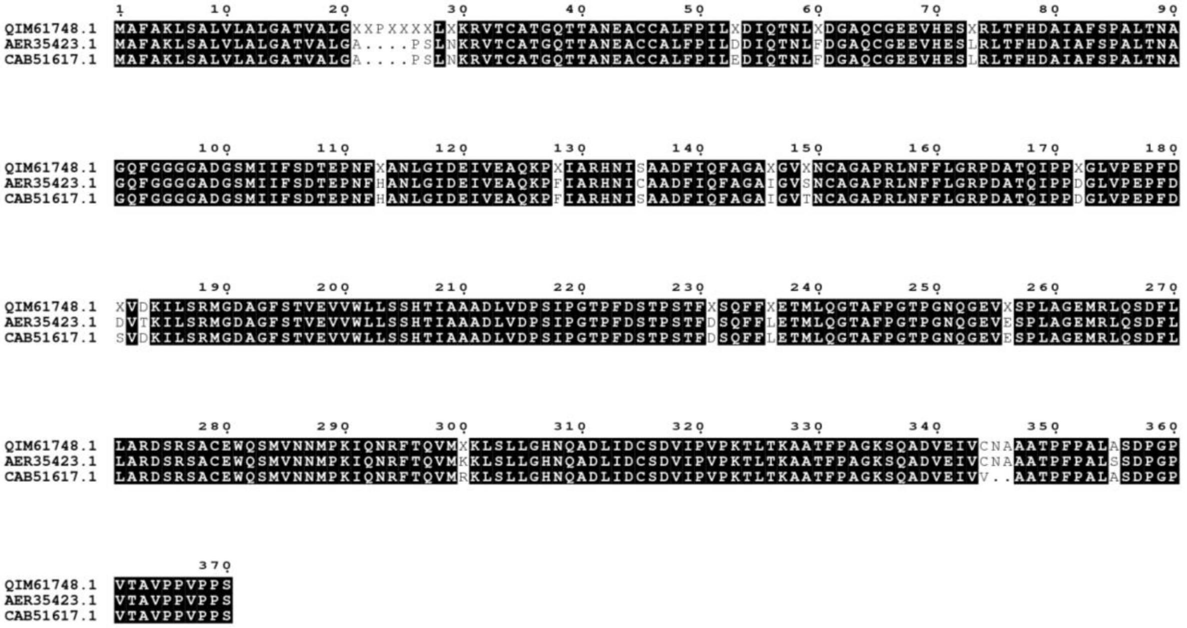
QIM61748.1
AER35423.1
CAB51617.1

b

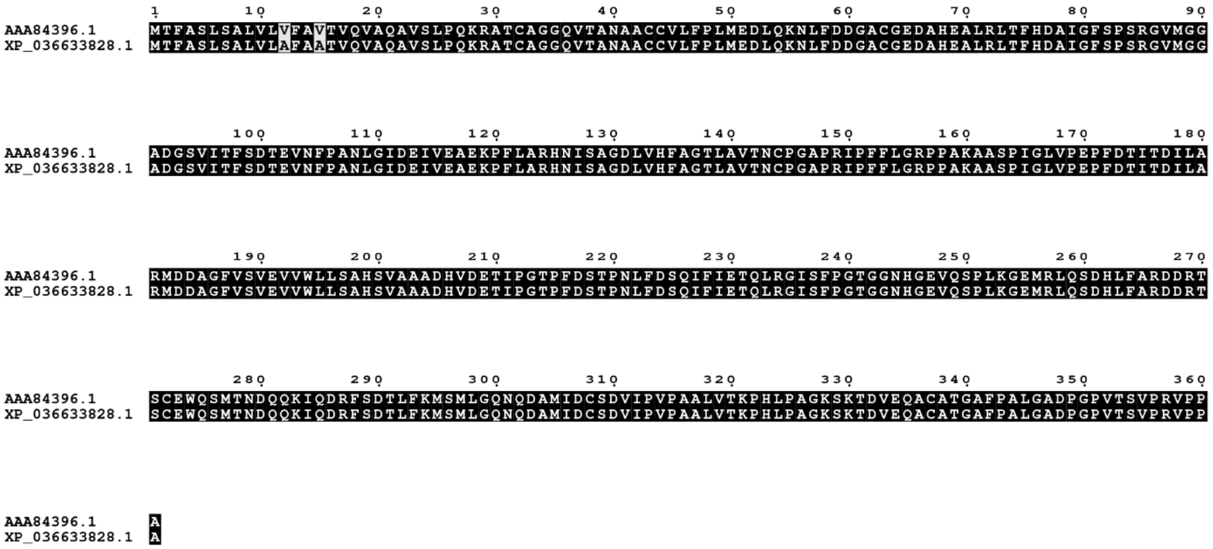

c

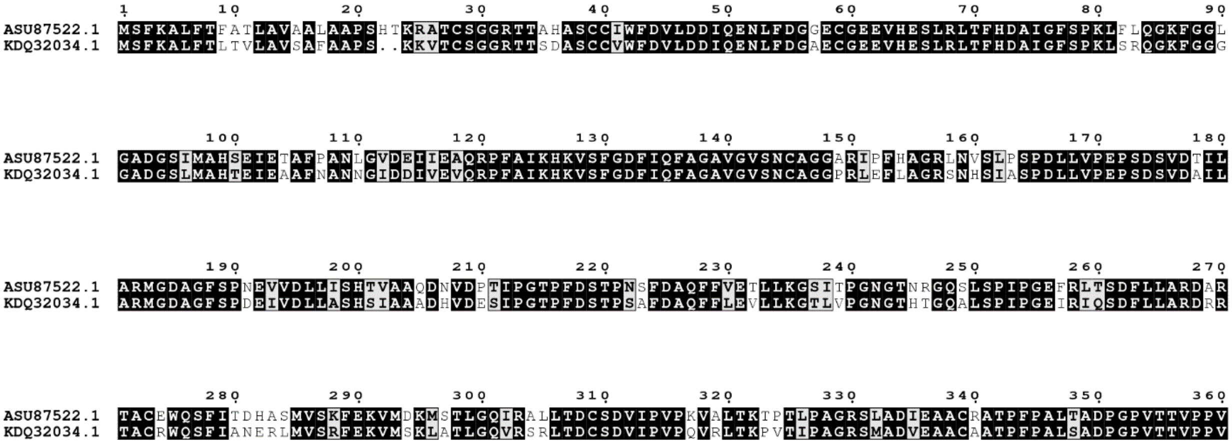

d

Supplementary Figure S3. Amino acid alignment of manganese peroxidases from *Pleorotus ostreatus*. a, b, c and d: different groups of manganese peroxidases. Highlighted amino acid in black: 100% of similarity among the amino acids; grey: 90–80%; white: similarity under 70%.
