# Supplementary material for: Assessing the Biodegradation of Vulcanised Rubber Particles by Fungi Using Genetic, Molecular and Surface Analysis
Source: Front Bioeng Biotechnol. 2021 Oct 18;9:761510. doi: 10.3389/fbioe.2021.761510 (PMC8558253; doi:10.3389/fbioe.2021.761510)
Supplement: Supplementary file 4 [file DataSheet1.PDF]

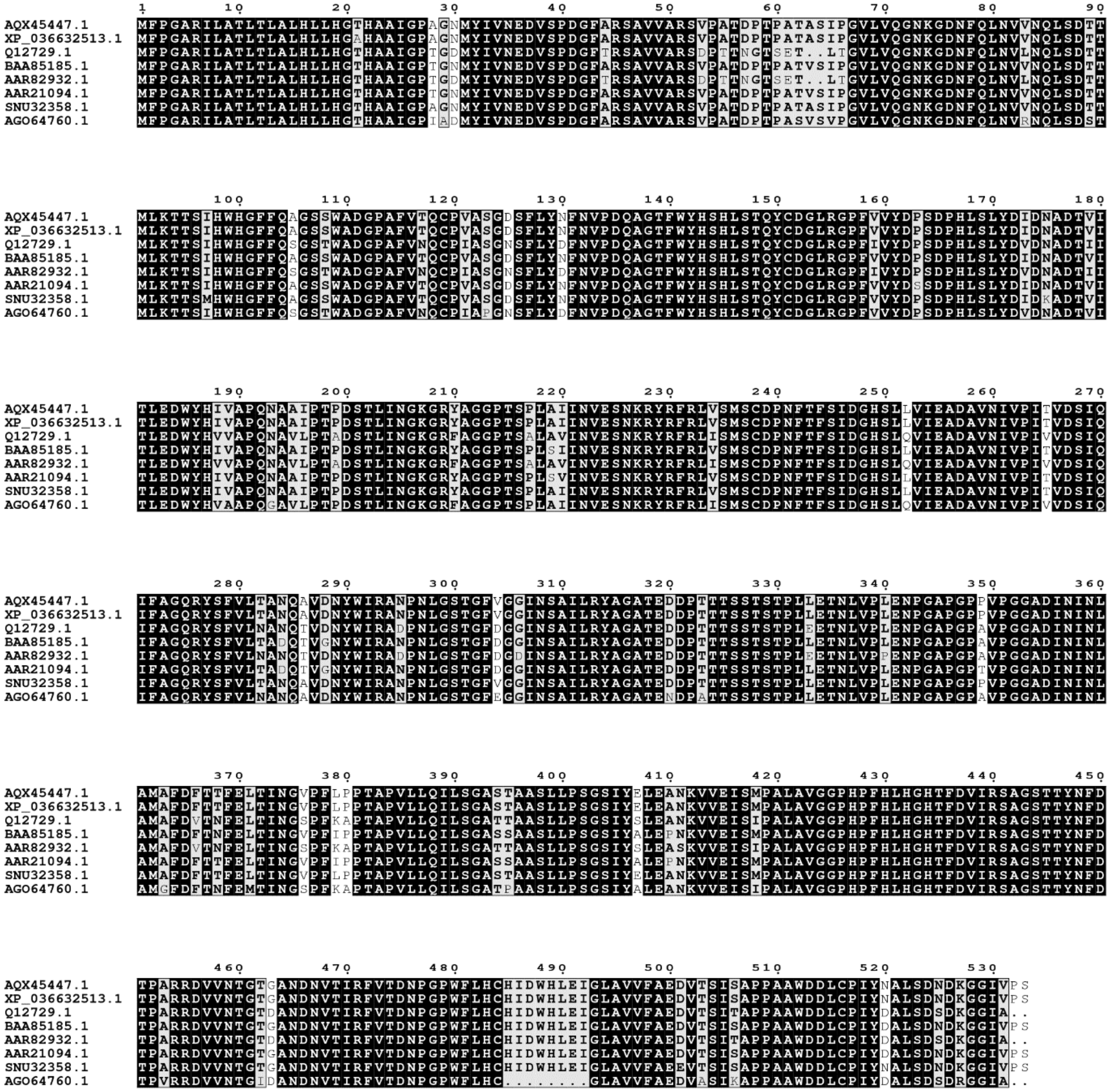
AQX45447.1
XP_036632513.1
Q12729.1
BAA85185.1
AAR82932.1
AAR21094.1
SNU32358.1
AGO64760.1

**a**

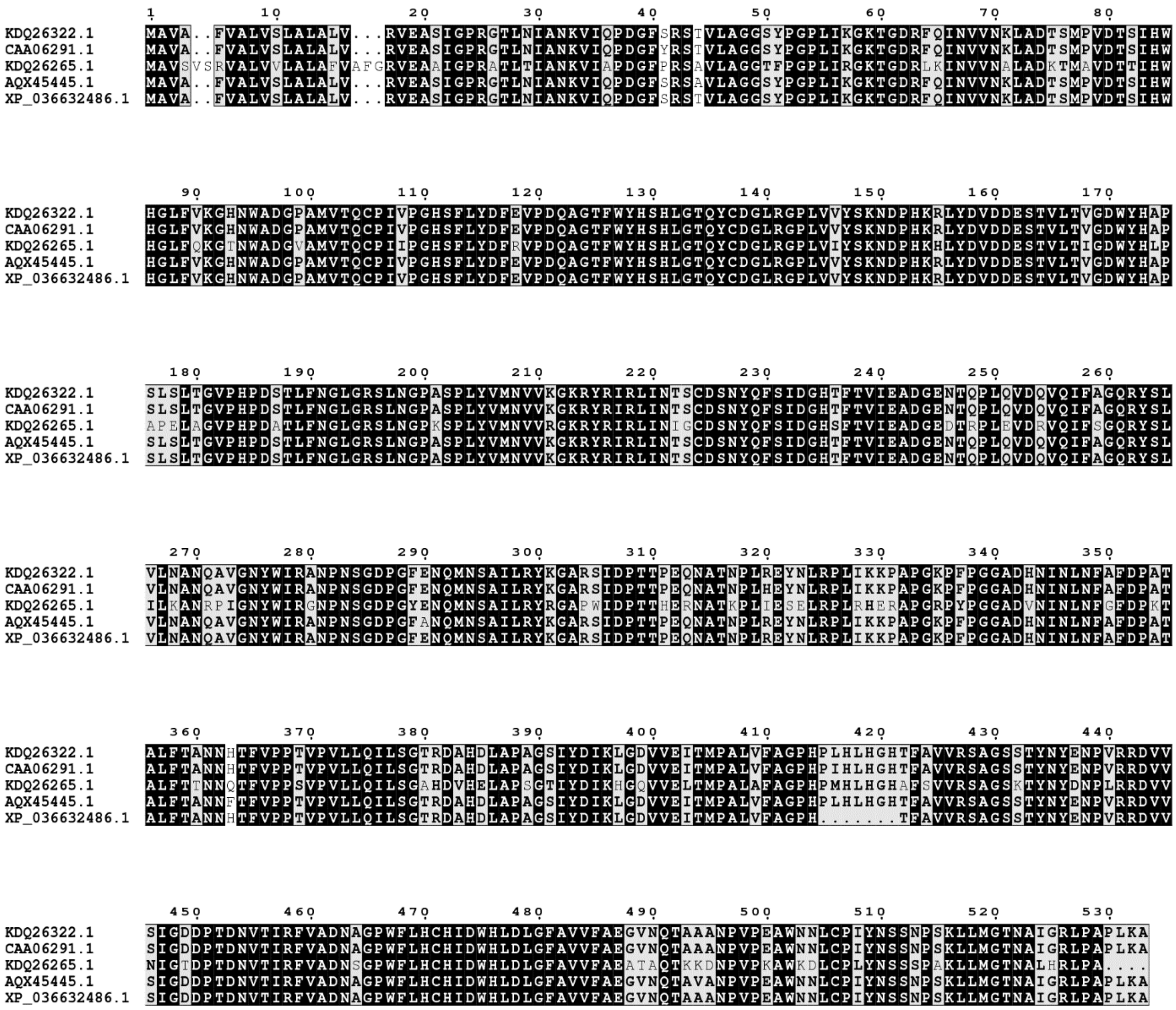
KDQ26322.1 MAVA..FVALVSLALALV...RVEASIGPRGTLNIANKVIQPDGFSRSTVLAGGSYPGPLIKGKTGDRFQINVVNKLADTSMPVDTSIHW
CAA06291.1 MAVA..FVALVSLALALV...RVEASIGPRGTLNIANKVIQPDGFYRSTVLAGGSYPGPLIKGKTGDRFQINVVNKLADTSMPVDTSIHW
KDQ26265.1 MAVSVSRVALVVLALAFVAFGRVEAAIGPRATLTIANKVIAPDGFPRSAVLAGGTFPGPLIRGKTGDRLKINVVNALADKTMAVDTTIHW
AQX45445.1 MAVA..FVALVSLALALV...RVEASIGPRGTLNIANKVIQPDGFSRSAVLAGGSYPGPLIKGKTGDRFQINVVNKLADTSMPVDTSIHW
XP_036632486.1 MAVA..FVALVSLALALV...RVEASIGPRGTLNIANKVIQPDGFSRSTVLAGGSYPGPLIKGKTGDRFQINVVNKLADTSMPVDTSIHW
KDQ26322.1 HGLFVKGHNWADGPAMVTQCPIVPGHSFLYDFEVPDQAGTFWYHSHLGTQYCDGLRGPLVVYSKNDPHKRLYDVDDESTVLTVGDWYHAP
CAA06291.1 HGLFVKGHNWADGPAMVTQCPIVPGHSFLYDFEVPDQAGTFWYHSHLGTQYCDGLRGPLVVYSKNDPHKRLYDVDDESTVLTVGDWYHAP
KDQ26265.1 HGLFQKGTNWADGVAMVTQCPIIPGHSFLYDFRVPDQAGTFWYHSHLGTQYCDGLRGPLVIYSKNDPHKHLYDVDDESTVLTIGDWYHLP
AQX45445.1 HGLFVKGHNWADGPAMVTQCPIVPGHSFLYDFEVPDQAGTFWYHSHLGTQYCDGLRGPLVVYSKNDPHKRLYDVDDESTVLTVGDWYHAP
XP_036632486.1 HGLFVKGHNWADGPAMVTQCPIVPGHSFLYDFEVPDQAGTFWYHSHLGTQYCDGLRGPLVVYSKNDPHKRLYDVDDESTVLTVGDWYHAP
KDQ26322.1 SLSLTGVPHPDSTLFNGLGRSLNGPASPLYVMNVVKGKRYRIRLINTSCDSNYQFSIDGHTFTVIEADGENTQPLQVDQVQIFAGQRYSL
CAA06291.1 SLSLTGVPHPDSTLFNGLGRSLNGPASPLYVMNVVKGKRYRIRLINTSCDSNYQFSIDGHTFTVIEADGENTQPLQVDQVQIFAGQRYSL
KDQ26265.1 APELAGVPHPDATLFNGLGRSLNGPKSPLYVMNVVRGKRYRIRLINIGCDSNYQFSIDGHSFTVIEADGEDTRPLEVDRVQIFSGQRYSL
AQX45445.1 SLSLTGVPHPDSTLFNGLGRSLNGPASPLYVMNVVKGKRYRIRLINTSCDSNYQFSIDGHTFTVIEADGENTQPLQVDQVQIFAGQRYSL
XP_036632486.1 SLSLTGVPHPDSTLFNGLGRSLNGPASPLYVMNVVKGKRYRIRLINTSCDSNYQFSIDGHTFTVIEADGENTQPLQVDQVQIFAGQRYSL
KDQ26322.1 VLNANQAVGNYWIRANPNSGDPGFENQMNSAILRYKGARSIDPTTPEQNATNPLREYNLRPLIKKPAPGKPFPGGADHNINLNFAFDPAT
CAA06291.1 VLNANQAVGNYWIRANPNSGDPGFENQMNSAILRYKGARSIDPTTPEQNATNPLHEYNLRPLIKKPAPGKPFPGGADHNINLNFAFDPAT
KDQ26265.1 ILKANRPIGNYWIRGNPNSGDPGYENQMNSAILRYRGAPWIDPTTHERNATKPLIESELRPLRHERAPGRPYPGGADVNINLNFGFDPKT
AQX45445.1 VLNANQAVGNYWIRANPNSGDPGFANQMNSAILRYKGARSIDPTTPEQNATNPLREYNLRPLIKKPAPGKPFPGGADHNINLNFAFDPAT
XP_036632486.1 VLNANQAVGNYWIRANPNSGDPGFENQMNSAILRYKGARSIDPTTPEQNATNPLREYNLRPLIKKPAPGKPFPGGADHNINLNFAFDPAT
KDQ26322.1 ALFTANNHTFVPPTVPVLLQILSGTRDAHDLAPAGSIYDIKLGDVVEITMPALVFAGPHPLHLHGHTFAVVRSAGSSTYNYENPVRRDVV
CAA06291.1 ALFTANNHTFVPPTVPVLLQILSGTRDAHDLAPAGSIYDIKLGDVVEITMPALVFAGPHPIHLHGHTFAVVRSAGSSTYNYENPVRRDVV
KDQ26265.1 ALFTTNNQTFVPPSVPVLLQILSGAHDVHELAPSGTIYDIKHGQVVELTMPALAFAGPHPMHLHGHAFSVVRSAGSKTYNYDNPLRRDVV
AQX45445.1 ALFTANNFTFVPPTVPVLLQILSGTRDAHDLAPAGSIYDIKLGDVVEITMPALVFAGPHPLHLHGHTFAVVRSAGSSTYNYENPVRRDVV
XP_036632486.1 ALFTANNHTFVPPTVPVLLQILSGTRDAHDLAPAGSIYDIKLGDVVEITMPALVFAGPH.......TFAVVRSAGSSTYNYENPVRRDVV
KDQ26322.1 SIGDDPTDNVTIRFVADNAGPWFLHCHIDWHLDLGFAVVFAEGVNQTAAANPVPEAWNNLCPIYNSSNPSKLLMGTNAIGRLPAPLKA
CAA06291.1 SIGDDPTDNVTIRFVADNAGPWFLHCHIDWHLDLGFAVVFAEGVNQTAAANPVPEAWNNLCPIYNSSNPSKLLMGTNAIGRLPAPLKA
KDQ26265.1 NIGTDPTDNVTIRFVADNSGPWFLHCHIDWHLDLGFAVVFAEATAQTKKDNPVPKAWKDLCPLYNSSSPAKLLMGTNALHRLPA....
AQX45445.1 SIGDDPTDNVTIRFVADNAGPWFLHCHIDWHLDLGFAVVFAEGVNQTAVANPVPEAWNNLCPIYNSSNPSKLLMGTNAIGRLPAPLKA
XP_036632486.1 SIGDDPTDNVTIRFVADNAGPWFLHCHIDWHLDLGFAVVFAEGVNQTAAANPVPEAWNNLCPIYNSSNPSKLLMGTNAIGRLPAPLKA

**b**

```
                 1        10        20        30        40        50        60        70        80        90
CAC69853.1       MVLSTKLAALVASLPFVLAATKKLDFHIRNDVVSPDGFERRAITVNGIFPGTPVILQKNDKVQINTINELTDPGMRRSTSIHWHGLFQHK
XP_036628446.1   MVLSTKLAALVASLPFVLAATKKLDFHIRNDVVSPDGFERRAITVNGIFPGTPVILQKNDKVQINTINELTDPGMRRSTSIHWHGLFQHK
KDQ24506.1       MVLSTKLAALVASLPFVLAATKKLDFHIRNDVVSPDGFERRAITVNGIFPGTPVILQKNDKVQINTINELTDPGMRRSTSIHWHGLFQHK

                         100       110       120       130       140       150       160       170       180
CAC69853.1       TSGMDGPSFVNQCPIPPNSTFLYDFDTAGQTGNYWYHSHLSTQYCDGLRGSFIVYDPNDPLKHLYDVDDESTIITLADWYHDLAPHAQNQ
XP_036628446.1   TSGMDGPSFVNQCPIPPNSTFLYDFDTAGQTGNYWYHSHLSTQYCDGLRGSFIVYDPNDPLKHLYDVDDESTIITLADWYHDLAPHAQNQ
KDQ24506.1       TSGMDGPSFVNQCPIPPNSTFLYDFDTAGQTGNYWYHSHLSTQYCDGLRGSFIVYDPNDPLKHLYDVDDESTIITLADWYHDLAPHAQNQ

                         190       200       210       220       230       240       250       260
CAC69853.1       FFQTGSVPIPDTGLINGVGRFKGGPLVPYAVINVEQGKRY.RFRLIQISCRPFFTFSIDNHTFDAIEFDGIEHDPTPAQNIDIYAAQRAS
XP_036628446.1   FFQTGSVPIPDTGLINGVGRFKGGPLVPYAVINVEQGKRYRRFRLIQISCRPFFTFSIDNHTFDAIEFDGIEHDPTPAQNIDIYAAQRAS
KDQ24506.1       FFQTGSVPIPDTGLINGVGRFKGGPLVPYAVINVEQGKRYSRFRLIQISCRPFFTFSIDNHTFDAIEFDGIEHDPTPAQNIDIYAAQRAS

               270       280       290       300       310       320       330       340       350
CAC69853.1       IIVHANQTIDNYWIRAPLTGGNPAGNPNLDISLIRAILRYKGAPAVEPTTVATTGGHKLNDAEMHPIAQEGPGNLGTGPPDMAITLNIAQ
XP_036628446.1   IIVHANQTIDNYWIRAPLTGGNPAGNPNLDISLIRAILRYKGAPAVEPTTVATTGGHKLNDADMHPIAQEGPGNLGTGPPDMAITLNIAQ
KDQ24506.1       IIVHANQTIDNYWIRAPLTGGNPAGNPNLDISLIRAILRYKGAPAVEPTTVATTGGHKLNDAEMHPIAQEGPGNLGTGPPDMAITLNIAQ

               360       370       380       390       400       410       420       430       440
CAC69853.1       PNPPFFDINGISYLSPSVPVLLQMLSGARKPQDFLPSEQVIILPANKLIEVSIPGAGAHPFHLHGHTFDIVRTSNSDVVNLVNPPRRDVL
XP_036628446.1   PNPPFFDINGISYLSPSVPVLLQMLSGARKPQDFLPSEQVIILPANKLIEVSIPGAGAHPFHLHGHTFDIVRTSNSDVVNLVNPPRRDVL
KDQ24506.1       PNPPFFDINGISYLSPSVPVLLQMLSGARKPQDFLPSEQVIILPANKLIEVSIPGAGAHPFHLHGHTFDIVRTSNSDVVNLVNPPRRDVL

               450       460       470       480       490       500       510       520
CAC69853.1       PINGGNTTFRFFSGNSGAWFLHCHIDWHLEAGLAVVFAERPAEVNEGEQAQIVTQDWRTLCPAYDGLAPEFQ
XP_036628446.1   PINGGNTTFRFFSGNSGAWFLHCHIDWHLEAGLAVVFAERPAEVNEGEQAQIVTQDWRTLCPAYDGLAPEFQ
KDQ24506.1       PINGGNTTFRFFSGNSGAWFLHCHIDWHLEAGLAVVFAERPAEVNEGEQAQIVTQDWRTLCPAYDGLAPEFQ
```

**C**

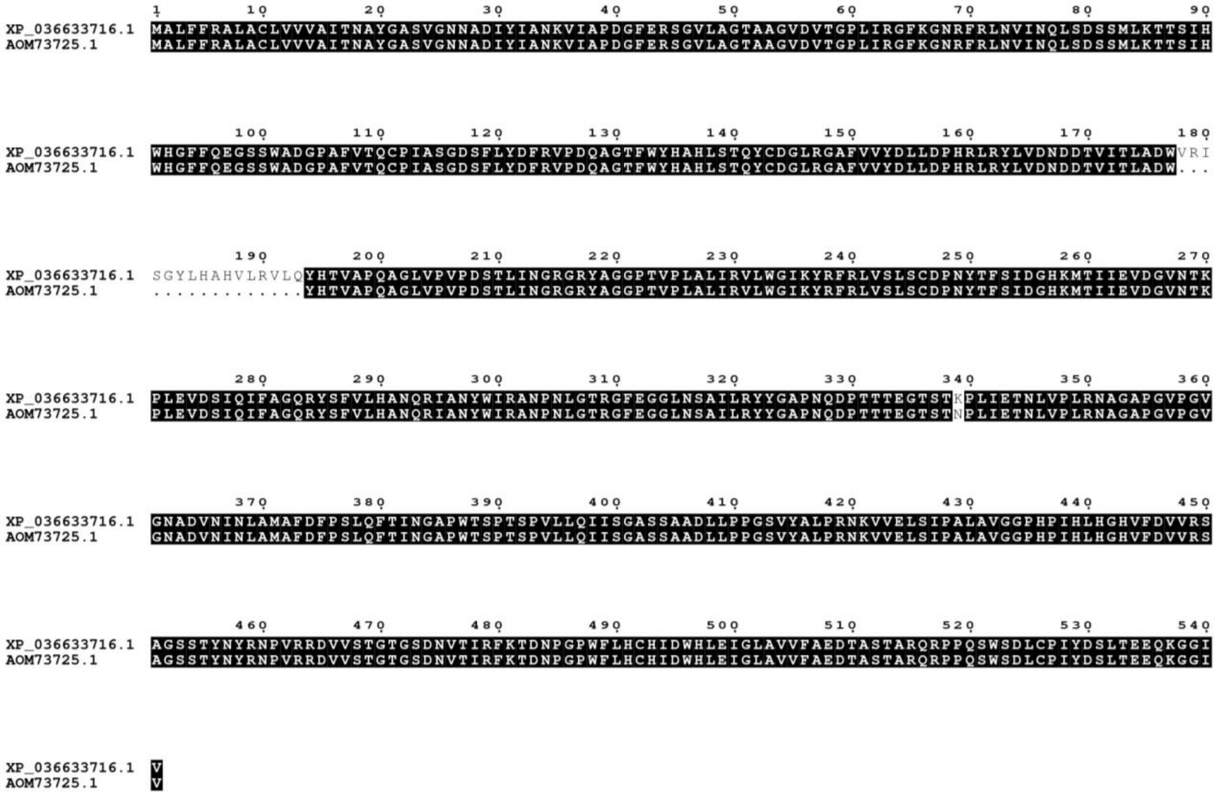


**d**

**Supplementary Figure S1.** Amino acid alignment of laccases from *Pleorotus ostreatus*. a, b, c and d: different groups of laccases. Highlighted amino acid in black: 100% of similarity among the amino acids; grey: 90–80%; white: similarity under 70%.
